# Evaluation of the performance of classification algorithms for XFEL single-particle imaging data

**DOI:** 10.1107/S2052252519001854

**Published:** 2019-02-28

**Authors:** Yingchen Shi, Ke Yin, Xuecheng Tai, Hasan DeMirci, Ahmad Hosseinizadeh, Brenda G. Hogue, Haoyuan Li, Abbas Ourmazd, Peter Schwander, Ivan A. Vartanyants, Chun Hong Yoon, Andrew Aquila, Haiguang Liu

**Affiliations:** a Department of Engineering Physics, Tsinghua University, 30 Shuangqing Rd, Haidian, Beijing 100084, People’s Republic of China; b Complex Systems Division, Beijing Computational Science Research Centre, 8 E Xibeiwang Rd, Haidian, Beijing 100193, People’s Republic of China; c Center for Mathematical Sciences, Huazhong University of Science and Technology, Wuhan, Hubei 430074, People’s Republic of China; d Department of Mathematics, University of Bergen, PO Box 7800, Bergen, 5020, Norway; e Biosciences Division, SLAC National Accelerator Laboratory, 2575 Sand Hill Road, Menlo Park, CA 94025, USA; f Stanford PULSE Institute, SLAC National Accelerator Laboratory, 2575 Sand Hill Road, Menlo Park, CA 94025, USA; g Department of Physics, University of Wisconsin–Milwaukee, Milwaukee, Wisconsin USA; h Biodesign Center for Immunotherapy, Vaccines, and Virotherapy, Biodesign Institute at Arizona State University, Tempe, 85287, USA; i Linac Coherent Light Source, SLAC National Accelerator Laboratory, 2575 Sand Hill Road, Menlo Park, CA 94025, USA; j Department of Physics, Stanford University, 450 Serra Mall, Stanford, CA 94305, USA; k Deutsches Elektronen-Synchrotron DESY, Notkestrasse 85, Hamburg, D-22607, Germany; l National Research Nuclear University MEPhI (Moscow Engineering Physics Institute), Kashirskoe shosse 31, Moscow, 115409, Russian Federation

**Keywords:** X-ray free-electron lasers (XFELs), single-particle imaging, classification algorithms, electron-density map reconstruction

## Abstract

Using X-ray free-electron lasers (XFELs), it is possible to determine three-dimensional structures of nanoscale particles using single-particle imaging methods. Classification algorithms are needed to sort out the single-particle diffraction patterns from the large amount of XFEL experimental data. However, different methods often yield inconsistent results. This study compared the performance of three classification algorithms: convolutional neural network, graph cut and diffusion map manifold embedding methods. The identified single-particle diffraction data of the PR772 virus particles were assembled in the three-dimensional Fourier space for real-space model reconstruction. The comparison showed that these three classification methods lead to different datasets and subsequently result in different electron density maps of the reconstructed models. Interestingly, the common dataset selected by these three methods improved the quality of the merged diffraction volume, as well as the resolutions of the reconstructed maps.

## Abstract 2

The performances of three image-classification algorithms were evaluated. The three classification methods lead to different datasets and subsequently result in different electron density maps of the reconstructed models.

## 1. Introduction   

The ultrashort and bright X-ray pulses from free-electron lasers (XFELs) make it possible to determine the structure of single particles or even single molecules. Femtosecond coherent X-ray pulses are used to take snapshots of individual particles before the samples are destroyed by the intense X-rays; this approach is known as ‘diffraction before destruction’ (Aquila *et al.*, 2015 ▸; Chapman *et al.*, 2006 ▸; Neutze *et al.*, 2000 ▸; Reddy *et al.*, 2017 ▸; Seibert *et al.*, 2011 ▸). In these experiments, reproducible particles (often assumed to be identical) in random orientations are injected into the radiation region and scattering signals of the particle can be collected by detectors for one orientation each time. Three-dimensional structure reconstruction requires a large number of scattering patterns from particles in random orientations in order to obtain sufficient sampling. The determination of electron density maps from raw datasets needs to undergo a procedure composed of single-particle scattering pattern classification, orientation recovery and phase retrieval. In this article, we focus on the classification of scattering patterns to find those patterns resulting from the scattering of single particles; specifically, single virus particles.

Because of the small interaction region for single-particle imaging (SPI), the probability of XFEL pulses hitting a sample particle is low; more than 98% of XFEL pulses miss their target particles and produce blank patterns with background noise or scattering data from solvent droplets. It is also possible for XFEL pulses to intercept more than one particle during one exposure, producing scattering patterns with inter-particle interference, often referred to as ‘multiple hits’, as opposed to the ‘single hits’ that are the scattering patterns from individual sample particles. The empty frames or data from multiple-particle scattering must be filtered out because they do not contain information that can be used for single-particle reconstruction. The procedure of identifying the scattering patterns is called ‘hit finding’. Several data reduction and analysis programs have been developed for hit finding, such as *Psocake* based on the *psana* framework (Damiani *et al.*, 2016 ▸), *CASS* (Foucar *et al.*, 2012 ▸) and *Cheetah* (Barty *et al.*, 2014 ▸). These programs are effective at filtering out blank patterns or weakly scattering objects (such as small water droplets) but the challenges in excluding multiple hits remain along with other more complicated cases. Therefore, advanced algorithms are needed to further classify the filtered data and identify a clean set of single-particle scattering patterns to improve data quality for structure recovery. Thus, in this work, the term ‘pattern identification’ is used for hit finding, or identifying the patterns with scattering signals without distinguishing the scattering sources; while the term ‘pattern classification’ is used for sorting the patterns of single sample particles or multiple particles. Here, three classification algorithms were applied to pattern classification.

Unsupervised computational methods were developed in recent years for SPI diffraction image classification, such as principal component analysis (PCA) with spectral clustering (Yoon *et al.*, 2011 ▸), diffusion map (DM) manifold embedding (Giannakis *et al.*, 2012 ▸; Schwander *et al.*, 2012 ▸) and particle-size filters determined via image autocorrelation functions (Andreasson *et al.*, 2014 ▸; Bobkov *et al.*, 2015 ▸). Pattern decomposition methods used in image processing and computer vision are also suitable for solving these kinds of problems, such as isomap embedding (Yoon, 2012 ▸) and *t*-distributed stochastic neighbour embedding (van der Maaten & Hinton, 2008 ▸). These methods are generally based on feature extraction and clustering in feature space. Features are composed of a small set of parameters that describe the most important characteristics of the original objects of interest. Clustering methods for general purposes are well developed at present, including *k*-means, spectral clustering and others. However, in many cases, these decomposition algorithms output different feature spaces when they are applied to experimental data of different samples and thus clustering methods must be designed in a problem-specific manner. Furthermore, prior knowledge about the data distribution in feature space may be needed to select the correct cluster that corresponds with the desired single-particle scattering patterns.

In this article, we introduce two supervised algorithms based on the convolutional neural network (CNN) and graph cut (GC) framework (Yin & Tai, 2018 ▸), then assess their performance in single-particle scattering data classification. The CNN method extracts features from data that are significant for distinguishing the classes by training the designed network against a labelled dataset and then sorting the data to the appropriate class based on the evaluation of the extracted features. The GC method utilizes the measures of data similarity to group the data in a similar manner to image partitioning (or image segmentation). For each classification application, the CNN method may need fine tuning of the network architecture, such as the number of layers or how these layers are connected. The GC method is based on the similarity measured using conventional metrics, such as the least-square difference among the original images, so it can be generalized to the classification of datasets for different samples without fine-tuning the parameters. Nonetheless, both CNN and GC require a training step for the method to learn the data properties so they depend on the quality of the training dataset. The dataset used in this study is from an X-ray scattering experiment conducted with LAMP instrument (Osipov *et al.*, 2018 ▸) at the AMO beamline of the Linac Coherent Light Source (LCLS) at the SLAC National Accelerator Laboratory. This dataset contains 64 511 scattering patterns identified by *Cheetah* and *Hummingbird* programs (Barty *et al.*, 2014 ▸; Daurer *et al.*, 2016 ▸) from millions of raw data frames. The data were obtained from the scattering of coliphage PR772, a DNA virus with an icosahedral capsid shell (Coetzee *et al.*, 1979 ▸). The two-dimensional diffraction patterns have resolutions of 11.6 nm at the edge and 8.3 nm at the corner of the detector, providing an oversampling rate of ∼40. The same dataset has been analysed by other groups from the single-particle imaging initiatives with different approaches (Aquila *et al.*, 2015 ▸; Hosseinizadeh *et al.*, 2017 ▸; Kurta *et al.*, 2017 ▸; Rose *et al.*, 2018 ▸). In this study, we compared the results of the classification methods and analysed the differences in the final reconstructed models from each individual dataset and the commonly selected dataset.

## 2. Methods   

### 2.1. Data preparation   

The CNN and GC methods are supervised classification approaches so a training dataset composed of manually labelled patterns is needed. Data preparation is critical for supervised methods, especially deep-learning methods, as the training datasets directly affect the outcomes of the trained model. Usually a large well-designed training set is required to yield a general classifier that can be used robustly. However, in XFEL experiments, it is very difficult to train a single CNN model for scattering patterns from different types of samples. One practical solution is to prepare training data for each type of sample. Considering the difficulties of manually labelling a large number of patterns, we labelled a small set of data composed of 200 randomly selected patterns from the original dataset deposited in the Coherent X-ray Imaging Data Bank (CXIDB). The training data had 79 single-particle scattering patterns and 121 patterns from other scatters such as water droplets, multiple particles or background noises, labelled as non-single-particle scattering patterns. We did not attempt to further classify the non-single-particle scattering patterns as the major goal was to distinguish the single-particle data from others.

**Figure 1:**
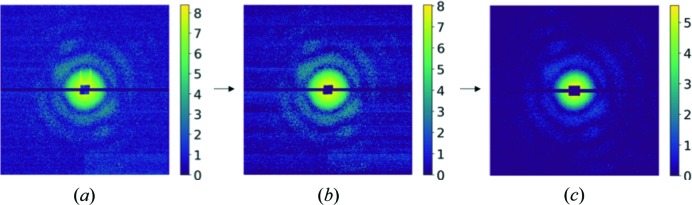
Pre-treatment of scattering patterns. (*a*) The original pattern, (*b*) after ‘bad’-pixel fixation using Friedel symmetry and (*c*) after photon-count conversion. The intensities are shown in logarithm scale to display the details. The apparent contrast difference between (*b*) and (*c*) is caused by the removal of weak signals (negative or analogue signals smaller than one photon were set to zero).

The scattering patterns were pre-processed before training or classification, as summarized in Fig. 1 ▸. The original scattering patterns were down-sampled by combining 4 × 4 pixels into a single pixel (Reddy *et al.*, 2017 ▸). Artefacts such as ‘bad’ pixels were fixed by using the values from their Friedel symmetric pixels and the analogue digital unit (ADU) values were converted to photon counts. The intensity values were then subjected to logarithm operations to enhance the features in regions with weaker signals. To avoid taking logarithm on zero values, the intensities were increased by 1.0 prior to the logarithm operation. Finally, the resulting patterns after the logarithm operation were scaled to have the same mean value. This helps to achieve a good balance of weighting factors through the resolution range of the compared images because a chi-square type of measurement is regularly used to quantify the data differences in CNN or GC methods. It should be noted that these processed patterns were only used as inputs for classification. The down-sampled data after ADU-photon conversion were used for orientation recovery and model reconstruction in the subsequent analysis.

### 2.2. Classification methods   

#### 2.2.1. CNN method   

The CNN used in this study is based on the sequential model. The functional components of a CNN model are composed of three types of layers: convolutional layer, pooling layer and dense layer. The convolutional layer executes a convolution operation by swiping over each pattern through kernel windows. The pooling layer executes a zooming operation. Here we use 2 × 2 max-pooling, where the input feature patterns are resized to one-quarter of their original size by choosing the maximum values of every 2 × 2 block of original patterns. The dense layer is comprised of a set of neurons where each neuron is fully connected to all of the neurons in the previous layer to form an* M* × *N* weighting matrix. In CNN, the dense layer should be implemented behind a flattened layer, which reshapes two-dimensional feature maps into a one-dimensional array of neurons.

The CNN optimizes the parameters used in non-linear transformations to reproduce known labels. The parameter space is designed to avoid overfitting. As our training set is small, the CNN model contains only three convolutional layers and one hidden dense layer. After down-sampling the original pattern to the size of 64 × 64 pixels, the whole CNN network contains 316 parameters. The number of model parameters is a little higher than the number of training samples (316 versus 200). To reduce the chance of overfitting and speed up parameter optimization, we randomly drop out a subset of neurons in both the convolutional layers and the dense layers (Srivastava *et al.*, 2014 ▸) and normalize each batch after every convolutional layer; thus, the mean and variance are 0 and 1, respectively. The CNN architecture is described in Section S1 of the Supporting information.

#### 2.2.2. GC method   

GC is an algorithm for semi-supervised clustering of high-dimensional data (Yin & Tai, 2018 ▸). In this method, the scattering patterns are modelled as vertices of a weighted graph with the weights defined as the similarity measurements of the connected vertices. Each vertex defined with intensity values (*x*) is only connected to a small number of the nearest vertices to make it a sparse graph. The similarity measure *w* between the two vertices 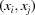 is defined as a radial basis function used in spectral clustering proposed in Zelnik-Manor & Perona (2004 ▸): 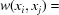 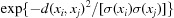, where the distance *d* is the Euclidean distance and 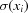 denotes the standard deviation of 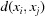 for fixed 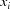.

The labelling function 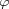 defined on each vertex takes values from [0,1], which can be interpreted as the probability of belonging to class 1 of single-particle patterns. The algorithm is proposed as a minimization of a convex functional of the labelling function. A variational method based on the Potts model is proposed for the partitioning of the graph, where each vertex is assigned a score between 0 and 1, indicating the likelihood of belonging to a specific cluster. More specifically, the convex functional consists of a data-fitting term (the so-called region force) based on an estimate of the probability of each vertex having a certain label and a regularization term that characterizes the total variation of the labelling function. This is formulated as the optimization problem
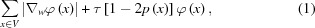
where 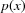 is the region force term modelling the prior probability of the vertex *x* belonging to class 1 given the already labelled data. 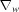 is the weighted gradient operator acting on the scalar-valued functions defined on the graph. The calculation of 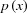 and 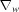 are explained in detail in S2. Once a solution 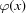 is obtained from the above minimization problem, the class of a vertex *x* is determined through a threshold process, that is, we choose some threshold value 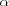 and set 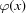 to 1 for 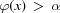 and to 0 otherwise. The term 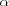 is chosen to be 0.5 unless specified otherwise. The integral of 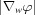 is then interpreted as the graph cut given the partition of the graph indicated by the labelling function 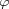. For more detailed information, see Yin & Tai (2018 ▸). The above convex minimization can be interpreted as a min-cut problem on the graph, whose dual is a max-flow problem, see Yuan *et al.* (2010 ▸). The solution to this problem can be obtained through a primal-dual algorithm. The details of this algorithm can be found in Yuan *et al.* (2010 ▸) with an adaptation to our application in Yin & Tai (2018 ▸). More details are also included in Section S2.

#### 2.2.3. DM manifold embedding   

DM manifold embedding is an eigenfunction-based feature extraction algorithm (Coifman & Lafon, 2006 ▸; Giannakis *et al.*, 2012 ▸). Assuming that every pixel stands for a dimension in a data manifold, the original patterns are embedded in a very high dimensional space. Similar to other embedding methods, DM is of type kernel-PCA and identifies principal components from the eigenvectors of an affinity matrix. Eigenvectors associated with the largest eigenvalues are used as features for classification. More specifically, the normalized graph Laplacian of the low-dimension manifold in feature space is used to calculate the likelihood of diffusion from the centre of the clusters. The procedures to calculate the DM kernel and the classification of scattering patterns are described in Section S3.

It has been shown that the eigenvectors from DM embedding can also represent orientation information of both cryo-EM and XFEL single-particle data (Hosseinizadeh *et al.*, 2014 ▸; Schwander *et al.*, 2012 ▸). The advantages of DM are very notable because of its simplicity of implementation and good orientation analysis performance. However, it has been shown that DM eigenvalues are continuously distributed in eigenspace (Hosseinizadeh *et al.*, 2015 ▸, 2014 ▸) making it difficult to do clustering using traditional algorithms without prior information about the manifold.

### 2.3. Phase retrieval   

Phase retrieval is an essential step to obtain real-space electron density maps and to compare the differences resulting from the classification methods. Two iterative phasing methods were used for phase retrieval: error reduction (Bauschke *et al.*, 2002 ▸) and difference map (Elser, 2003 ▸). Each phasing cycle contains 100 iterations of error-reduction phasing, 200 iterations of difference-map phasing and an additional 200 iterations of the error-reduction-phasing processes. The support region was updated by setting it to consist of 2000 voxels with highest intensities in the phased model at each iteration. The volume of the support region is estimated from the particle size and the oversampling rate of the input data. For every dataset, 40 independent phasing retrievals were carried out to obtain the averaged model after alignments as the final output models. The program is modified from the three-dimensional phasing program by Andrew Morgan and collaborators (Morgan, 2016 ▸). The phase-retrieval transfer functions (PRTFs) were calculated to evaluate the model resolutions,
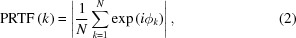
where *N* is the number of independent phasing results and 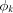 is the phase angle of the *k*th phase retrieval.

A convergence value was used to describe the difference between the current model and the one obtained in the previous iteration [equation (3)]. The difference between the calculated intensities and the input intensities is noted as the modulus error [equation (4)].
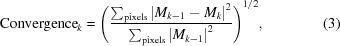


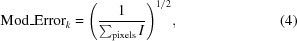
where *k* is the *k*th iteration, 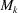 is the retrieved reciprocal model and *I* is the merged scattering intensity using the expansion–maximization–compression (EMC) algorithm im­plemented in *Dragonfly* (Ayyer *et al.*, 2016 ▸; Loh & Elser, 2009 ▸).

### 2.4. Testing dataset   

The dataset used for testing in this study was downloaded from the CXIDB (Maia, 2012 ▸). The data (CXIDB 58) were collected from experiment amo86615 carried out at the LCLS, the XFEL facility at the SLAC National Accelerator Laboratory. For a detailed description of the dataset and classification results using the DM manifold embedding approach refer to the work by Reddy *et al.* (2017 ▸). There are 64 511 patterns containing significant scattering signals, 14 772 of which were selected as single hits using the DM method (see Section S7).

## 3. Results   

We applied two supervised methods described in the *Methods* section[Sec sec2] to the same set of scattering data from the PR772 virus particles. The classification results were compared with the previously published results and the common subset from these three datasets was identified. The computing speed was evaluated to compare the analysis throughput. The single-particle datasets obtained from these three methods were compared from the perspectives of the reduced one-dimensional radial profiles and the merged three-dimensional diffraction volumes. Furthermore, phase retrievals were performed for each merged diffraction volume to investigate the real-space electron density maps resulting from each dataset.

### 3.1. Classification results   

Both the CNN and GC output scores between 0 and 1 to describe the likelihood of being single-particle diffraction patterns, and 0.5 was used as the cutoff to label the outcome classes. A validation dataset containing 500 manually labelled patterns was used to evaluate the accuracy of the trained models by computing the true positive rate at a cutoff of 0.5. CNN has a prediction accuracy of 83.8%, and the accuracies for GC and DM methods are 84.2 and 80.8%, respectively. It is notable that mistakes in the manually selected training/validation data could not be completely avoided; thus, these values are not the comparison results against the ground truth. The CNN and GC methods were tested using simulation data that are composed of scattering patterns from single particles and multiple particles [see examples in Figs. S8(*a*) and S8(*c*)]. The accuracy rates for both CNN and GC methods are above 96% for simulation dataset classification.

**Figure 2:**
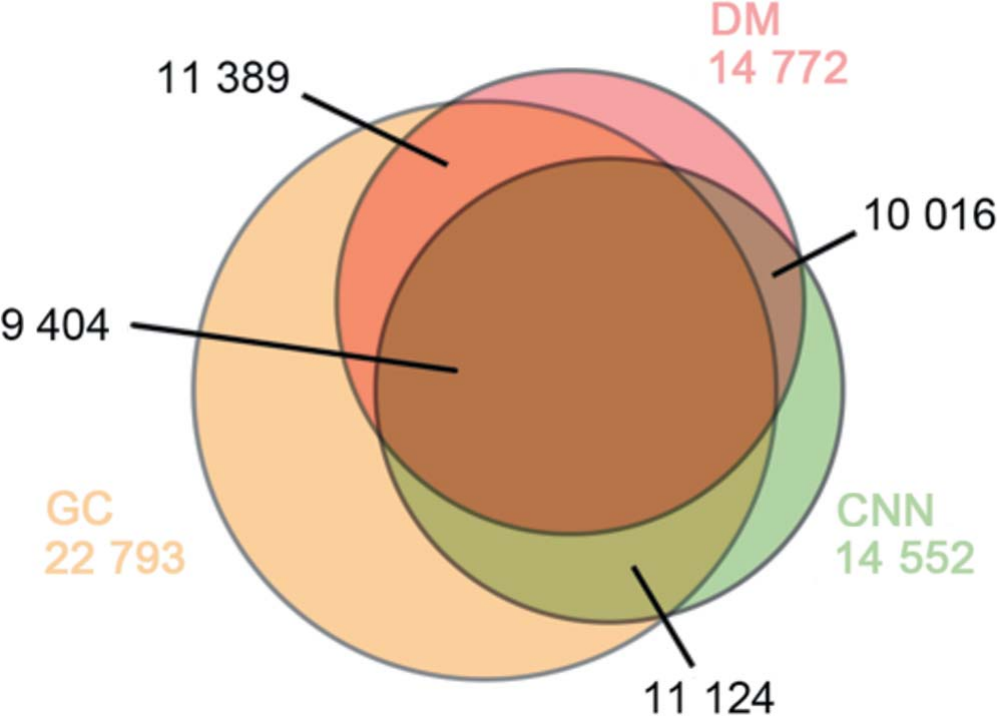
Venn diagram for the three sets of single-particle scattering patterns. There are 10 016 commonly selected patterns between the CNN and DM, 11 124 patterns between the GC and CNN, and 11 389 patterns between the GC and DM. A total of 9 404 patterns were tagged as single-particle scattering patterns by all three methods.

Specifically, the CNN selected 14 552 patterns as single hits, while the GC provided 22 793 single hits. In the published dataset selected using the DM method, there were 14 772 single hits. The fiducial number and event time associated with each pattern were used to identify the consensus and the differences in the classification results, which are summarized in a Venn diagram (Fig. 2 ▸). The common subset of selections resulting from the three methods is composed of 9 404 patterns. This implies that each method utilizes different properties of the patterns for the classification. The largest overlap occurs between the sets selected using the GC and DM methods, which contain 11 389 patterns in common.

As shown in Table 1 ▸, all three methods require a similar amount of computing time for the classification of this particular dataset. The model training time is included for the CNN method. CNN and DM are implemented using *Python* [CNN uses the *Theano* package (The Theano Development Team *et al.*, 2016 ▸) to build neural network architecture], while GC is a *MATLAB* program. The CNN used one CPU core for job management and most computing was performed using one NVIDIA K80 GPU.

### 3.2. Averaged intensity radial profiles   

**Figure 3:**
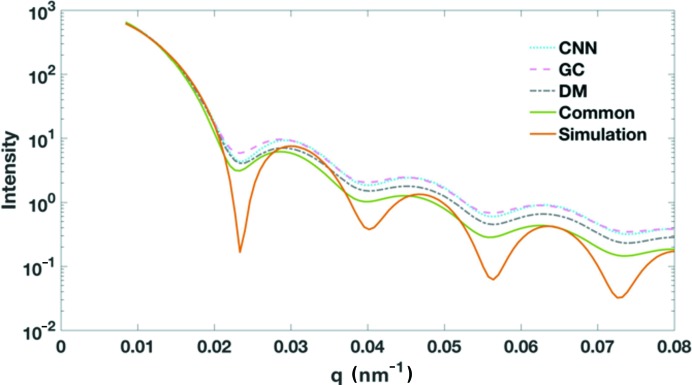
Averaged intensity radial profiles. Here *q* is calculated by 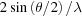, where 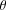 is the scattering angle and 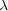 is the wavelength of the X-rays. The profiles are overlaid by the matching intensities at the low-*q* region.

A virtual small-angle X-ray scattering (SAXS) pattern and one-dimensional profile can be generated from the selected single-particle scattering patterns. Because of the limited numbers of patterns in each selected subset, the corresponding one-dimensional radial profile can be treated only as an approximation of the conventional SAXS profile, which is a summed contribution of the scattering signals from a very large number of particles. The three radial profiles exhibit similar intensity distributions as shown in Fig. 3 ▸. The positions of intensity minima in the three profiles are very consistent. The most evident divergence of the three curves occurs beyond *q* ≃ 0.022 nm^−1^, where the signals from the GC dataset are more similar to the CNN dataset. The common subset composed of patterns selected by all three methods is also converted to a one-dimensional radial intensity profile. This common subset has a radial profile with a faster decreasing trend, especially in the high-*q* region (*q* ≃ 0.022 nm^−1^). Because a measured SAXS profile for PR772 was not immediately available for comparison, we computed a simulated SAXS profile from an icosahedron model that approximates the PR772 particles. Although the solid model could not capture the electron density differences between the protein capsid and the interior DNA molecules, the SAXS profile can still serve as a reference to compare with the four radial profiles. As shown in Fig. 3 ▸, the overall profiles are very similar and the profile from the common dataset has better agreement in the high-resolution region to that of the icosahedron model.

### 3.3. Orientation recovery and merging   

**Figure 4:**
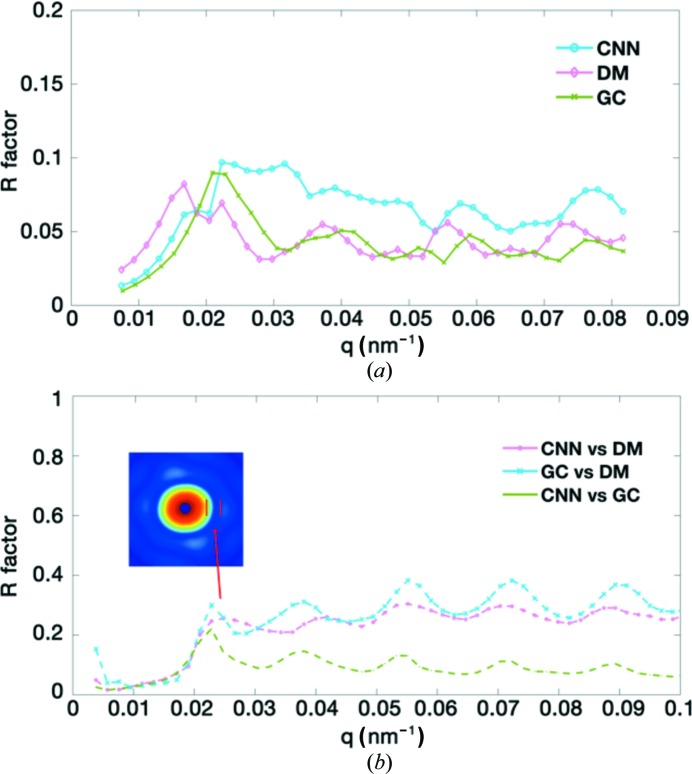
Self-consistency and cross-comparison of the merged results from the three datasets. Here *q* is calculated in the same way as in Fig. 3 ▸. The two graphs are (*a*) the *R* factors between the independent merged results from the same dataset and (*b*) the *R* factors for the merged results from the different datasets.

We applied the EMC algorithm implemented in *Dragonfly* to recover the orientations and merge the intensity to three-dimensional reciprocal space (Ayyer *et al.*, 2016 ▸; Loh & Elser, 2009 ▸). Because the EMC algorithm merges the two-dimensional scattering patterns to the diffraction volume iteratively from the random initial models, independent reconstructions for the same dataset may have some differences. To evaluate the consistency of the merged results, two independent reconstructions from the random initial models with the same control parameters were carried out for each selected dataset. The evaluation of the *R* factor after model alignment (Fig. 4 ▸) shows high consistency between the independent reconstructions for all three datasets. The overall *R* factors of the merged results for the datasets selected using the CNN, GC and DM are 0.076, 0.062 and 0.074, respectively. The results from the CNN dataset have the largest *R* factors; yet, the values are still under 10%, indicating good self-consistency of the merged results with the independent starting models. The merged diffraction volumes also have good correspondence with individual single-particle scattering patterns, see Fig. S5 for two representative patterns compared with their best matched central slices from the merged data.

The comparisons of the merged intensities in the three-dimensional diffraction volumes from the different datasets were measured using *R* factors at discrete resolution shells [Fig. 4 ▸(*b*)]. There are significant *R*-factor peaks among the merged results for the three datasets. These regions correspond to the intensity minimums as shown in the inset of Fig. 4 ▸(*b*) (see also Fig. 3 ▸).

**Figure 5:**
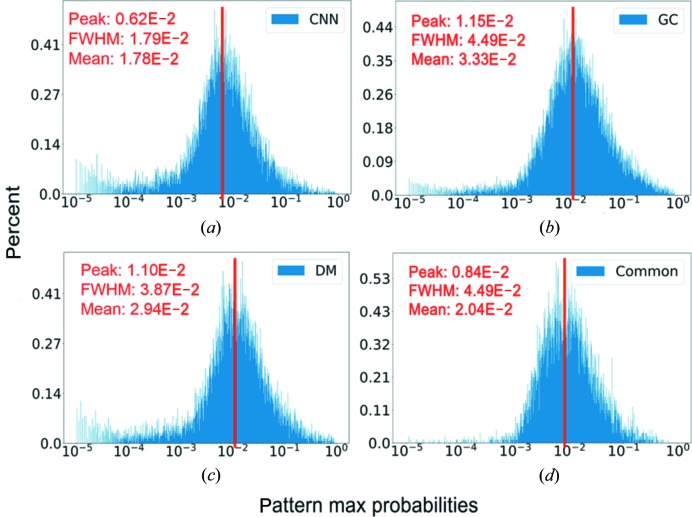
The distributions of the largest probability for each pattern. For randomly sampled orientations, the expected probability should be approximately 2 × 10^−5^. For the CNN (*a*) , the GC (*b*) and the DM (*d*) methods, the percentage of patterns whose largest probabilities are smaller than 10^−4^ are 7.19, 2.62 and 6.76%, respectively. In comparison, the common dataset (*d*) has 9404 patterns, 28 of which have the largest probability smaller than 10^−4^ (∼0.3%).

As the EMC algorithm calculates the probabilities of patterns at 50 100 different spatial orientations (including in-plane rotations), we selected the most likely orientations that had the largest probabilities (*p*_max_) for each pattern and studied the distributions of *p*_max_ values (Fig. 5 ▸). For a pattern composed of random numbers, the expectation of probability is approximately 2 × 10^−5^ (1/50100). The dataset selected using the GC method has a probability distribution centred around 0.0115 with a relatively small number of ‘bad’ patterns (∼2.62%) whose orientations are uncertain (probability <10^−4^). In the dataset selected using the CNN and DM methods, the orientations for a relatively larger number of patterns are not well determined (7.19 and 6.76%, respectively) as indicated by the population with low probabilities in Figs. 5 ▸(*a*) and 5(*c*). Quite interestingly, we found that the common dataset has far fewer patterns whose orientations are uncertain (28 out of 9404 patterns have their *p*_max_ < 10^−4^). From Fig. 5 ▸, we can also see that only a small fraction of patterns have their *p*_max_ values < 10^−3^ in the common dataset. This strongly suggests that the combined selection power of the three methods helps the exclusion of ‘bad’ diffraction patterns.

The orientation distributions of the patterns were also investigated. For each pattern, we selected ten orientations with the highest probabilities. The in-plane rotations were not explicitly considered in the distribution analysis so the orientations could be displayed on the surface of a sphere. The probabilities of each pattern being at each of the ten most likely orientations were then summed to the corresponding orientations, which were mapped to the spherical surface. Then we obtained the probability for the patterns identified at each given orientation as shown in Fig. S4. The large variation of probabilities indicated by the size of the dots suggests that orientation preferences exist in this dataset. In order to rule out that this orientation anisotropy was introduced during the merging or the orientation distribution analysis, we carried out the orientation recovery using the same procedure on a simulated dataset composed of 10 000 single-particle patterns sampled at random orientations. The orientation distribution for the simulated data is shown in Fig. S8(*b*), from which no significant orientation bias could be observed. The variations in probabilities for the simulated dataset are much smaller than those of experimental data. Since all four datasets exhibited similar orientation bias, it is plausible to be the nature of the PR772 dataset.

### 3.4. Phase retrieval   

**Figure 6:**
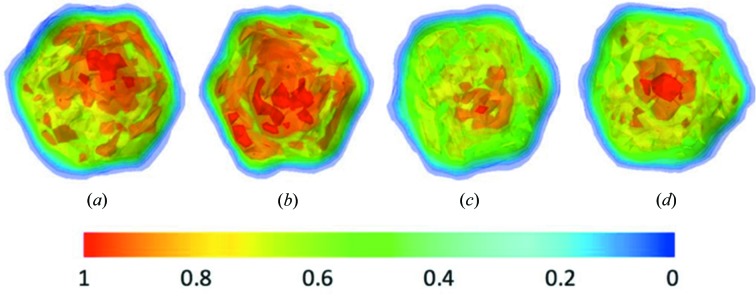
Contour display of the retrieved electron density maps. The maps for the datasets selected using the (*a*) CNN, (*b*) GC, (*c*) DM and (*d*) common dataset.

Using the phase-retrieval algorithm described in the *Methods* section[Sec sec2], the real-space models were reconstructed for the merged data from patterns selected using the three methods. The retrieved density maps are shown in Fig. 6 ▸. All of the reconstructed models display icosahedral symmetry for the virus capsid. However, the genetic materials (DNA molecules) enclosed by the capsid are not located in the very centre of the reconstructed models, coloured in red because of this higher electron density compared with protein molecules. As observed in the electron density maps, the CNN and DM datasets yielded models with DNA molecules shifted toward one of the fivefold vertices. Interestingly, the reconstructed model from the common subset exhibited the least shift of the enclosed DNA molecules [Fig. 6 ▸(*d*)]. The reconstructed map similarities were assessed using the Fourier shell correlation (FSC). All pairwise comparisons were carried out using *EMAN2* package (Tang *et al.*, 2007 ▸). Using a cutoff value of 0.5, the consistency levels between maps reconstructed from four datasets were summarized in Table 2 ▸ (see Fig. S9 for FSC curves). In general, the maps are consistent with each other to about 10.0 nm resolution, except for the case of the CNN dataset which yielded a map that exhibited significant inconsistency at about 11.5 nm resolution compared with the maps from GC or DM datasets.

Using 1/*e*, where *e* is the Euler number *e* = 2.71828, as the PRTF cutoff, model resolutions from the datasets selected using the CNN, GC and DM methods are 11.6, 9.2 and 11.8 nm, respectively (see Fig. S6). Although the commonly selected dataset contains only 9404 patterns, the reconstructed model has the highest resolution of 8.8 nm based on the same criteria (nearly to the resolution corresponding with the corner of the detector, 8.5 nm). This might be because the common dataset is composed of data from particles with fewer variations.

**Figure 7:**
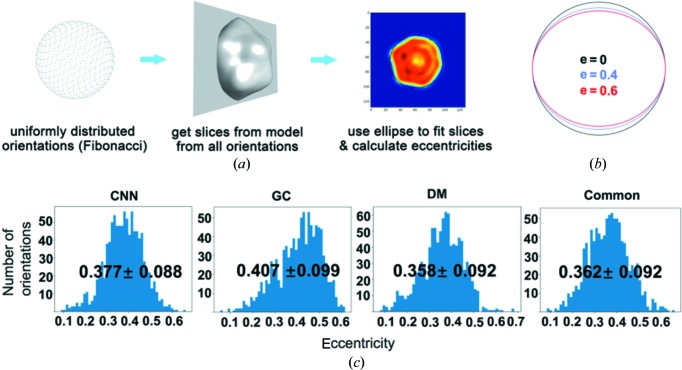
The shape analysis of the reconstructed maps. (*a*) An illustration of the cross-section slicing procedure: a Fibonacci sampling algorithm was used to select the direction of the planes that pass through the model centre. (*b*) Ellipses with three eccentricity values to guide the understanding of the deviation from a perfect circle. (*c*) The distribution of eccentricity values of map cross-sections for four reconstructed maps.

The shapes of the reconstructed virus particles were examined using the eccentricity by fitting the cross-sections with ellipses [Fig. 7 ▸(*a*)]. The cross-section planes were uniformly selected based on Fibonacci sampling on a sphere surface. The parameters for the optimally fitted ellipses were then used to compute the eccentricity [*e* = *c*/*a*, where *c* is the distance between foci and *a* is the length of the major axis, see Fig. 7 ▸(*b*) for examples]. The distributions of the eccentricity are summarized in Fig. 7 ▸(*c*) for the four reconstructed maps. The results indicate that the reconstructed models have clear deviation from a perfect sphere or icosahedron. The mean values of the eccentricity are in the range between 0.358 and 0.407 for the four models. This is consistent with a discovery in a recent report, where the authors found that the PR772 virus particles deviated from ideal icosahedral symmetry (Rose *et al.*, 2018 ▸).

## 4. Discussion and conclusions   

XFEL single-particle imaging is an alternative method for determining structures of large biological particles and has potential to visualize conformational changes of three-dimensional nanoscale objects. Advanced data-analysis methods are critical for the development of the XFEL single-particle imaging method. Herein we evaluated the performance of three classification methods and their impact on the reconstructed models.

The supervised classification approach requires a training dataset labelled by experts or other methods. In this study, the performance of the algorithm is acceptable even with a small training dataset. It is possible to manually label 200 single-particle scattering patterns with a good signal-to-noise ratio within a few minutes, so these methods can potentially be used for real-time data classification during experiments. In practice, it is always better to include more labelled data as training dataset to improve the accuracy and robustness of the classification methods.

Because of the conformation heterogeneity of the PR772 samples, the electron density inside the virus capsid of the reconstructed models from each individual classification method exhibited a clear deviation toward one of the fivefold vertices. It is interesting to observe that the common dataset leads to a model with a higher resolution and more centralized genetic materials [Fig. 6 ▸(*d*)]. Arguably, the ‘bad’ patterns are partially responsible for the lower resolution of individual datasets. For those ‘bad’ patterns whose orientations are not confidently recovered (*p*_max_ < 10^−4^, see Fig. 5 ▸), the signals contribute to the background and the inclusion of such patterns might affect the final model resolution. This indicates that the data quality and sample heterogeneity issue can be improved by combining several classification methods. In other words, a group decision based on a properly designed voting system may outperform individual methods.

A recent study revealed a conformational landscape of PR772, where a rearrangement of the distribution of the viral content could be observed by conformational analysis of XFEL data (Hosseinizadeh *et al.*, 2017 ▸). PR772 is a member of the *Tectiviridae* virus family and is similar to the prototype member, PRD1. During infection and also upon sample storage, it has been directly observed by electron microscopy that the PRD1 inner lipid membrane containing the DNA genome changes its icosahedral form and produces a proteolipid tube from a fivefold portal of the outer protein capsid, which results in the release of the viral genome (Peralta *et al.*, 2013 ▸; Santos-Pérez *et al.*, 2017 ▸). The differences in the phased models shown in Fig. 6 ▸ might be attributed to the bias of each dataset towards different conformational states. On the other hand, the common subset formed by the consensus of three methods might be less biased towards any particular conformational state. It may therefore be possible to use the consensus subset to determine the most populated state (often the ground state), serving as the first step for studying the conformational changes using model-based approaches. For example, using the model obtained from the common subset as the starting point, a series of models could be generated using structure perturbation or dynamic simulation methods. Then each generated model could be used as a reference to classify the experimental data. This model-based data classification approach could be improved iteratively by refining the initial models with the classified data.

In summary, we presented two supervised image-classification methods based on CNN and GC algorithms to identify scattering patterns resulting from the single particles. The performances and outcomes were compared against the published dataset selected using the DM manifold embedding method. Although the structure of PR772 virus is not known from an independent study to allow direct comparison, the cross-comparisons among the four datasets reveal interesting results. The commonly selected dataset contains far fewer ‘bad’ patterns whose orientations could not be recovered to high confidence levels. Furthermore, the phase-retrieval results revealed that the common dataset yielded a reconstructed model with higher resolutions. The enclosed DNA of PR772 is found to locate closer to the centre of the virus in the reconstructed model from the common dataset, in contrast to the other three reconstructed models from individually selected datasets. Given the rapid development in cryo-EM single-particle imaging methods, we hope to see the high-resolution structure of PR772, which will serve as the ground truth to assess the quality of these four datasets.

## Supplementary Material

The supplementary information for the method, extra figures . DOI: 10.1107/S2052252519001854/cw5020sup1.docx

The supplementary information for the method, extra figures . DOI: 10.1107/S2052252519001854/cw5020sup1.pdf

## Figures and Tables

**Table 1:** Computing-speed comparison

Algorithm	Hardware	Time
CNN	K80 GPU	∼5 min
GC	Xeon CPU (ten cores)	∼15 min
DM	Xeon CPU (ten cores)	∼20 min

**Table 2:** The consistency levels between the reconstructed maps from four datasets

Model A	Model B	*q* value at cutoff of 0.5 nm^−1^	Real-space resolution (nm)
CNN	DM	0.087	11.5
CNN	GC	0.087	11.5
DM	GC	0.102	9.8
CNN	Common	0.097	10.3
DM	Common	0.097	10.3
GC	Common	0.100	10.0
