# Supplementary material for: Evaluation of the performance of classification algorithms for XFEL single-particle imaging data
Source: IUCrJ. 2019 Feb 28;6(Pt 2):331–40. doi: 10.1107/S2052252519001854 (PMC6400180; doi:10.1107/S2052252519001854)
Supplement: Supplementary file 2 [file m-06-00331-sup1.pdf]

IUCrJ

Volume 6 (2019)

Supporting information for article:

Evaluation of the performance of classification algorithms for XFEL single-particle imaging data

Yingchen Shi, Ke Yin, Xuecheng Tai, Hasan DeMirci, Ahmad Hosseinizadeh, Brenda G. Hogue, Haoyuan Li, Abbas Ourmazd, Peter Schwander, Ivan A. Vartanyants, Chun Hong Yoon, Andrew Aquila and Haiguang Liu

S1. Convolutional neural network

The architecture of the convolutional neural network used in this study is shown in Figure S1. Binary cross-entropy and adaptive moment estimation (Adam) (Kingma & Ba, 2014) are used as a loss function and optimisation algorithm. Our program is established on the Keras framework with a Theano backend.

Based on the architecture in Figure S1, the number of parameters in CNN can be calculated as below.

Parameters in convolutional layers (Conv2D):

$$5 \times 5 \times 5 + 3 \times 3 \times 3 + 2 \times 3 \times 3 = 170$$

Output x and y dimensions of the last Max-polling layer:

$$\frac{\frac{\frac{64-(5-1)}{2}-(3-1)}{2}-(3-1)}{2} = 6$$

and parameters in Dense(2) layer:

$$(2 \times 6 \times 6) \times 2 = 144$$

Parameters in Dense(1) layer:

$$2 \times 1 = 2$$

As a result, CNN contains 170+144+2=316 parameters.

S2. Graph cut model

The construction of the sparse graph for the data and the computation of the adjacency matrix W is the most time-consuming part of the implementation of the GC algorithm. We used the MATLAB software package VLFeat (Vedaldi & Fulkerson, 2010) to construct the k-nearest neighbour graph for the dataset ($k = 5$ in this application), and the adjacency matrix W was computed accordingly. For simplicity, we refer to the gradient operator ∇_W defined on the graph as the symbol ∇. More exactly, let $u(x)$ be a function defined on the vertices of the graph G. Given vertex x_i and one of its neighbouring vertices x_j, the gradient of u at x_i in the direction of x_i to x_j is denoted by

$$\nabla u(x_i)(x_j) = w_{ij}\left(u(x_j) - u(x_i)\right).$$

Thus, $u(x_i)$ is a sparse N-dimensional vector with number of nonzero entries equal to the number of neighbours of x_i. By stacking all of the $\nabla u(x_i)$ in rows, we obtain ∇u as the $N \times N$ sparse matrix. Accordingly, we can define a “flow” variable q on the graph in the same dimension of ∇u, where a row, for example $q(x_i)$, denotes the flow from the vertex x_i to every vertex in the graph. The divergence operator (div) is defined as the adjoint operator to the gradient operator (∇), usually defined on a flow on the graph such that $divq$ is an N-dimensional vector. The prior probability $p(x)$ for vertices x of the graph models the conditional probability of x belonging to the single-particle

patterns given the set S_1 of the single-particle patterns and the set S_0 of the non-single-particle patterns. We use the following definition for $p(x)$:

$$p(x) = \frac{\frac{1}{|S_1|}\sum_{x_1 \in S_1} d(x, x_1)}{\frac{1}{|S_0|}\sum_{x_0 \in S_0} d(x, x_0) + \frac{1}{|S_1|}\sum_{x_1 \in S_1} d(x, x_1)},$$

where $d(x_i, x_j) = \frac{(w_{ij}^{(2)})^2}{w_{ii}^{(2)} w_{jj}^{(2)}}$. Here $w_{ij}^{(2)}$ is the (i, j) entry of the matrix W^2 (W to the second power).

The pseudo code for the GC algorithm is listed in Figure S2. In each iteration of the whole loop, there is one evaluation of the gradient of the labelling function φ and one evaluation of the divergence of the flow q, which take up most of the computation cost. In addition, there is one projection onto the infinity-norm ball ($\Pi_{||q||_\infty \le 1}$) that restricts each row of q to be unit vector and one projection onto the set Δ where each entry is on the interval [0,1].

S3. Diffusion map manifold embedding

Figure S3 shows the pseudo code of DM method, and the source code is available at https://github.com/haoyuanli93/DiffusionMap . For CXIDB 58, the first 3 components of the eigenvectors are used for clustering, where $(\Phi_1, \Phi_2, \Phi_3) = (-0.75,0,0)$ is recognised as the most ideal single-particle diffraction pattern.

S4. Calculation method of orientation distributions

The orientation distribution of the merged dataset is calculated as equation below, where P_r is the total probability of the rth orientation and p_{ir} is the probability of the rth orientation for the ith pattern. In this study, we only used the top 10 orientations with the highest probabilities for every pattern.

$$P_r = \sum_i p_{ir}$$

S5. Orientation recovery using simulation data

10,000 diffraction patterns were simulated from a solid icosahedron using randomly sampled Euler angles, shown in Figure S8a. The orientation recovery on this simulation data were carried out using the Dragonfly program, and the recovered orientation distribution is shown in Figure S8b. Although there are some fluctuations in the distributions, the is no belt-like distribution observed in Figure S4. Furthermore, the fluctuation levels are much smaller in the simulation dataset, compared to uneven distributions in the PR772 data. Simulation source code is available at https://github.com/LiuLab-CSRC/spipy/blob/examples/spipy/simulate/sim_adu.py.

S6. Data and source code

The raw dataset, training patterns, source code and classification results in this work are available at https://pr772-doc.readthedocs.io/en/latest/.

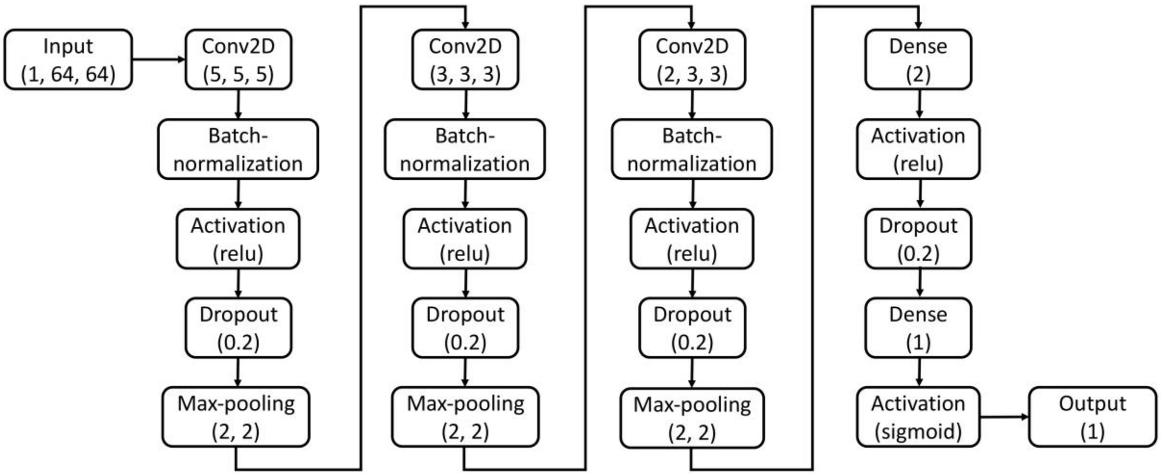

Figure S1 CNN architecture. The parameters inside the bracket of the convolutional layer are the output channels, the kernel size in the x axis, and the kernel size in the y axis, respectively. The parameter inside the bracket of the dense layer is the number of neurons.

Algorithm 1 Graph-cut based model for image classification

Require: prior probability p, weighted adjacency matrix W for the graph, $\{\beta_l = 0.2l\}$, $\{\gamma_l = 0.2/(1+0.1l)\}$, τ

Ensure: ϕ

function GC
- $l = 0$,
- **while** "not converged" **do**
  - calculate $\nabla\phi$,
  - $q = \Pi_{\|q\|_\infty \le 1}(q - \beta_l \nabla\phi)$,
  - calculate div$q$,
  - $\phi = \Pi_\Delta(\phi - \gamma_l(\mathrm{div}q + \tau(1-2p)))$,
  - $l = l + 1$,
- **end while**
- **return** ϕ.

end function

Figure S2 Pseudo code of graph cut (GC) model for image classification.

Algorithm 2 Diffusion map manifold embedding

Require: patterns p_i, number of patterns s, Gaussian width ε, number of closest neighbours N, α, number of components nEig

Return: $s \times nEig$ eigenvectors of diffusion map $eigVec$

function DM
- $D_{ij} = \|p_i - p_j\|^2, i, j = 1 \dots s$
- $K_{ij} = \exp(-D_{ij}/\varepsilon^2)$
- keep the highest N values of each row in K_{ij} and get K_{ij}'
- $K_{ij}' = \max(K_{ij}', K_{ij}'^T)$
- $K_{ij}'' = \left(\frac{K_{ij}'}{sum(K_{ij}', 1) * sum(K_{ij}', 2)}\right)^\alpha$
- diagonal matrix $W_{ii} = \sum_{j=1}^{s} K_{ij}''$
- kernel matrix $L = W^{-1/2} K'' W^{-1/2}$
- calculate eigenvectors of L and sort by eigenvalues
- $eigVec$ = the first nEig dimensions of each eigenvector
- **return** $eigVec$

end function

Figure S3 Pseudo code of diffusion map (DM) manifold embedding.

(a)

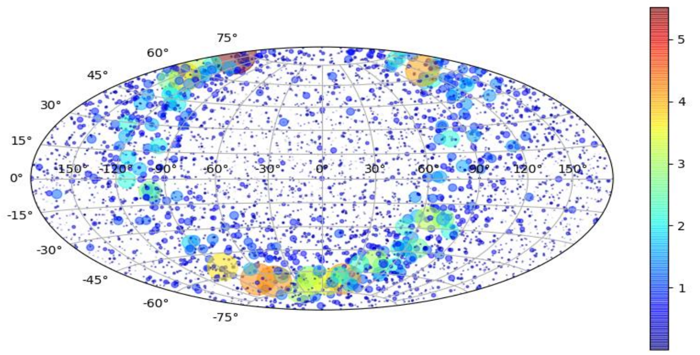
75°
60°
45°
30°
15°
0°
-15°
-30°
-45°
-60°
-75°
-150° -120° -90° -60° -30° 0° 30° 60° 90° 120° 150°
5
4
3
2
1

(b)

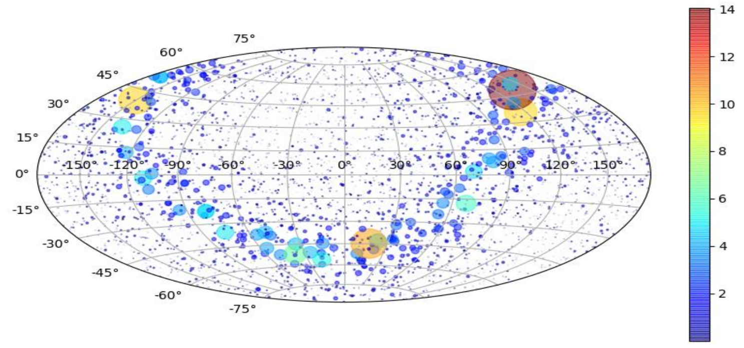
75°
60°
45°
30°
15°
0°
-15°
-30°
-45°
-60°
-75°
-150° -120° -90° -60° -30° 0° 30° 60° 90° 120° 150°
14
12
10
8
6
4
2

(c)

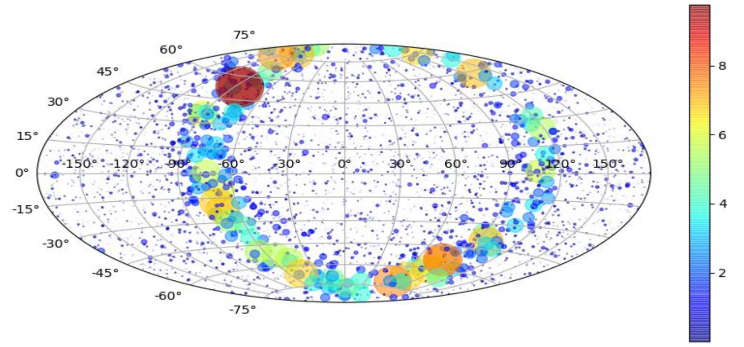
75°
60°
45°
30°
15°
0°
-15°
-30°
-45°
-60°
-75°
-150° -120° -90° -60° -30° 0° 30° 60° 90° 120° 150°
8
6
4
2

(d)

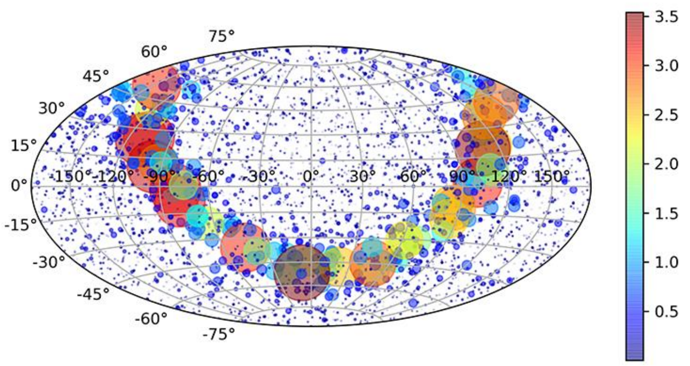

(e)

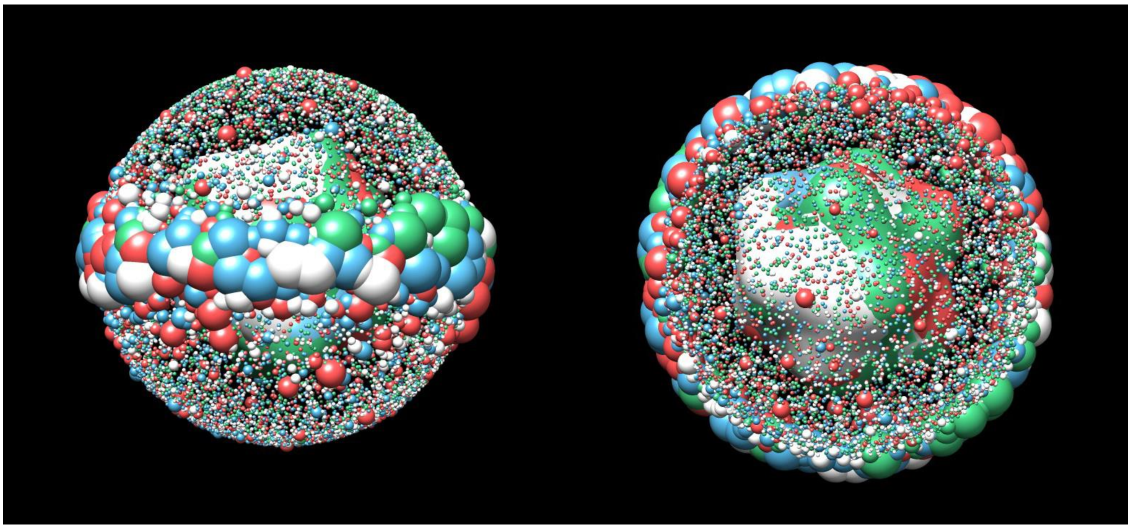

Figure S4 Orientation distributions. Hammer-Aitoff projection of the orientation distribution for the datasets from the three classification methods, CNN (a), GC (b), DM (c) and common (d) datasets. The colour bar displays the values of the probabilities, which are proportional to the radius of the circles in the figure. (e) The reconstructed model from the merged data is superposed, revealing that the most preferred orientation is around a belt. Colour codes: CNN (red), GC (white), DM (green) and common (blue) datasets.

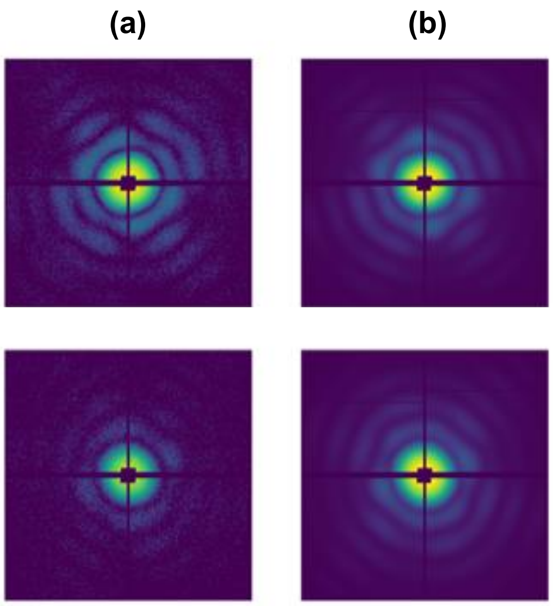

Figure S5 Representative examples of single scattering patterns compared against the slices from the merged model of CNN dataset. (a) two scattering patterns; (b) the best matched central slices to the patterns in (a). The same cross mask was applied to guide the visual comparison, the experimental patterns are down-sampled and the slices correspond to the same parameters.

(a)

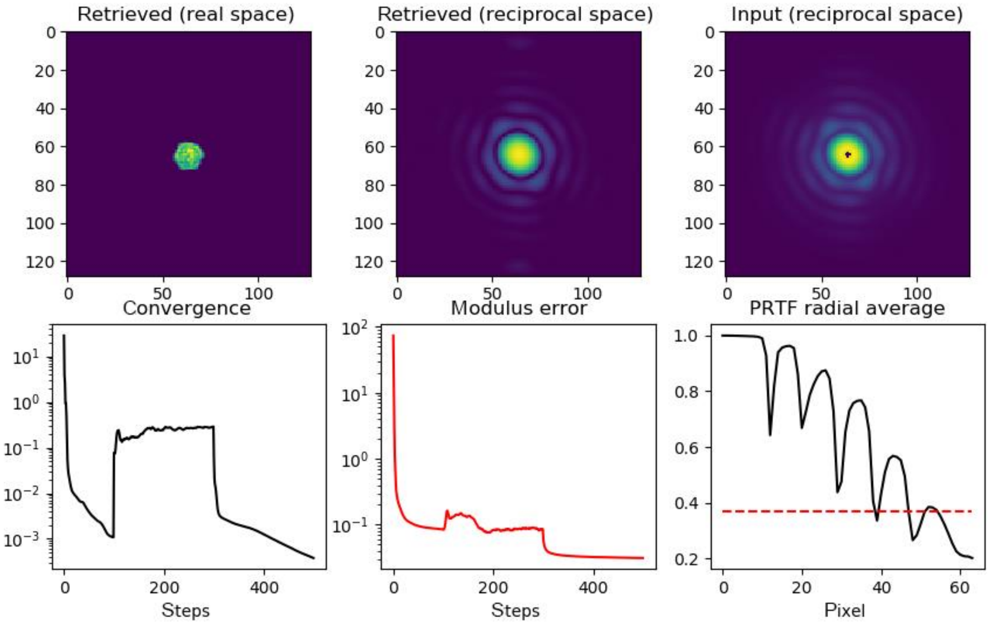

(b)

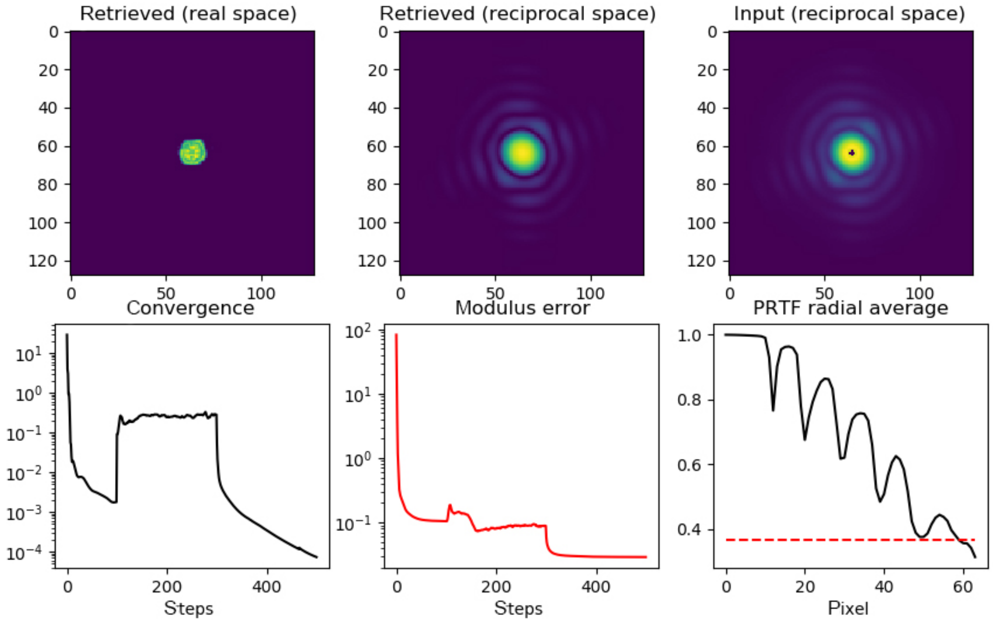
Retrieved (real space)
Retrieved (reciprocal space)
Input (reciprocal space)
Convergence
Modulus error
PRTF radial average
Steps
Steps
Pixel

(c)

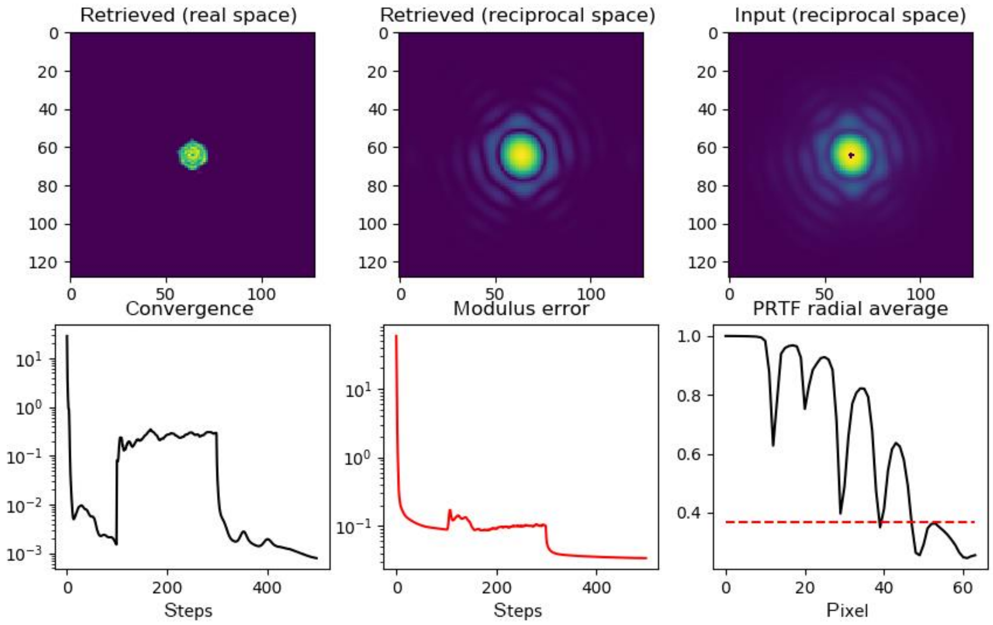
Retrieved (real space)
Retrieved (reciprocal space)
Input (reciprocal space)
Convergence
Modulus error
PRTF radial average
Steps
Steps
Pixel

(d)

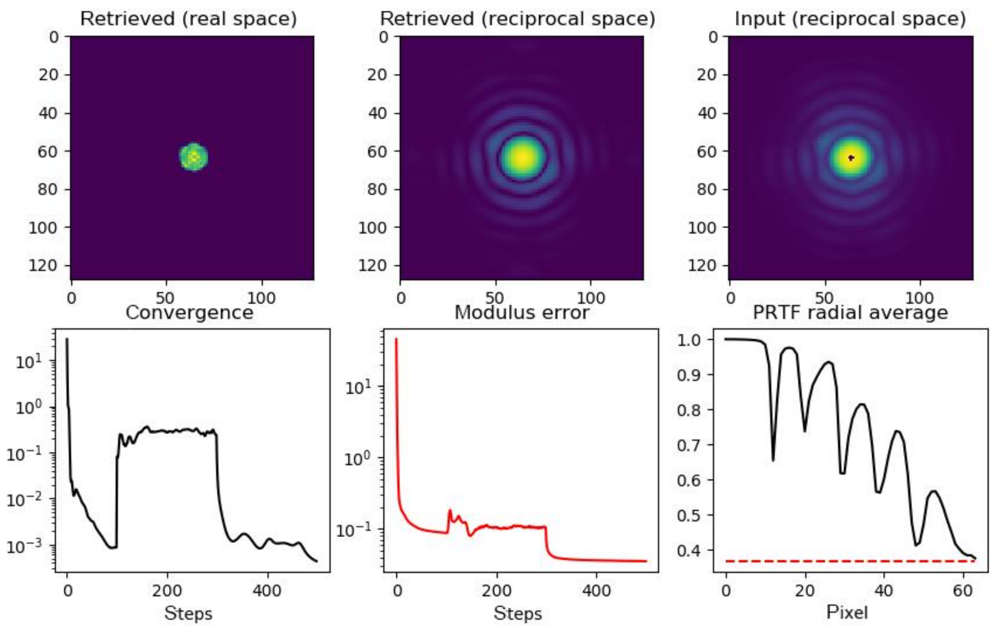

Figure S6 Phase retrieval results. Representative phase retrieval processes for (a) CNN, (b) GC, (c) DM methods, and (d) common datasets. The red dashed lines in the PRTF figure represent the 1/e cut-off, which is used to calculate the model resolutions. The middle panels show slices in the YZ plane of the reciprocal space to compare the input and retrieved intensities. The pixel size is 0.00185 nm^{-1}.

(a)

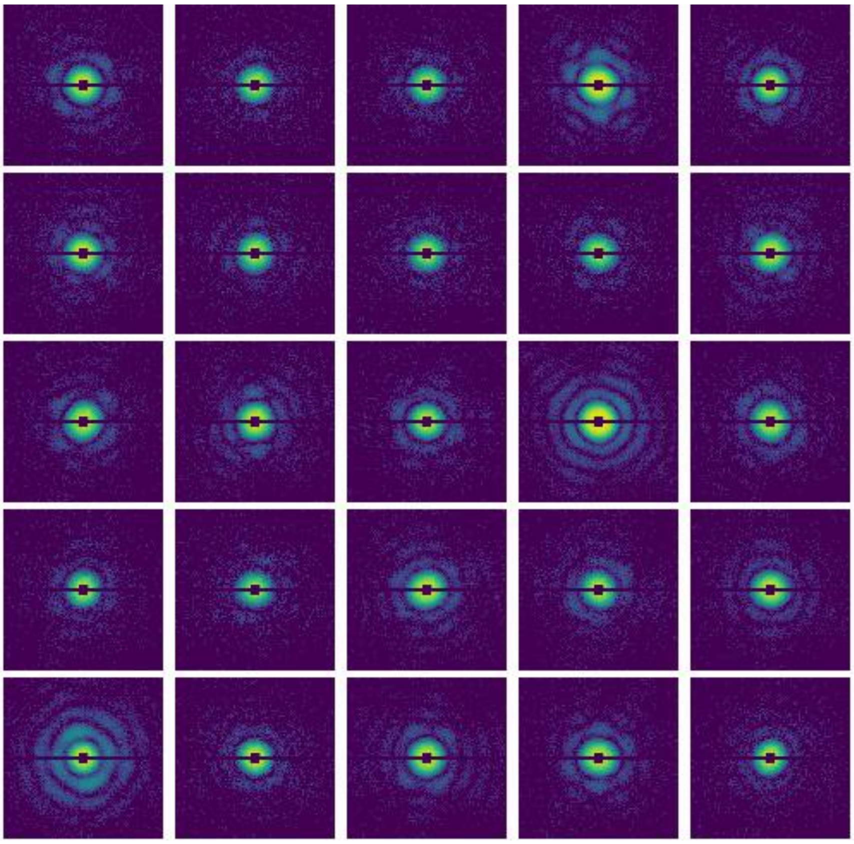

(b)

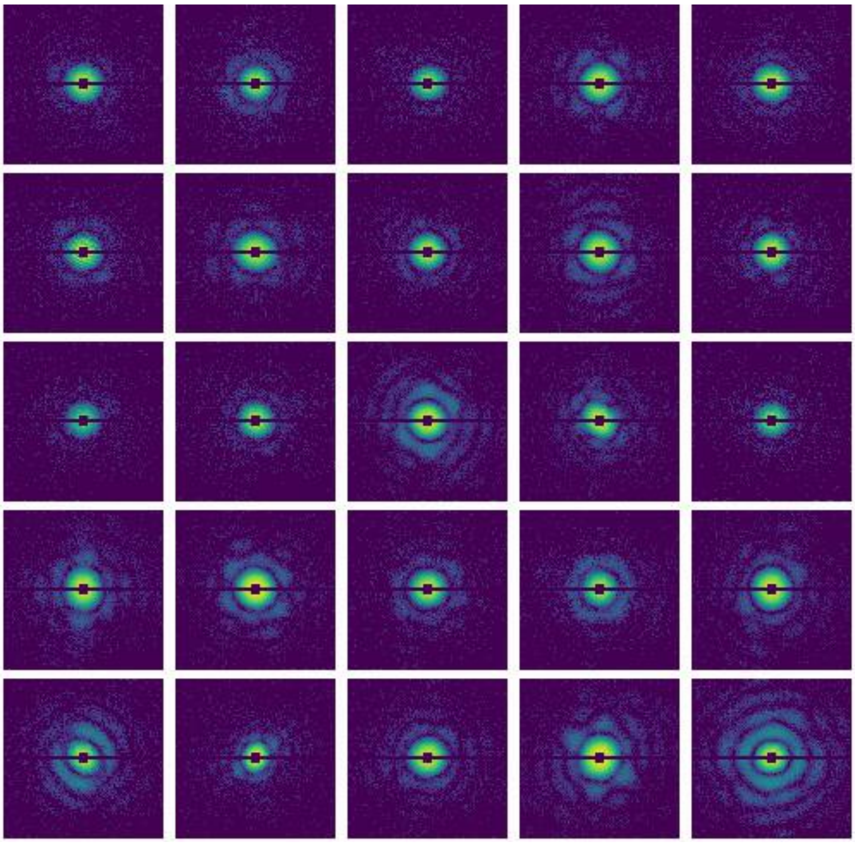

(c)

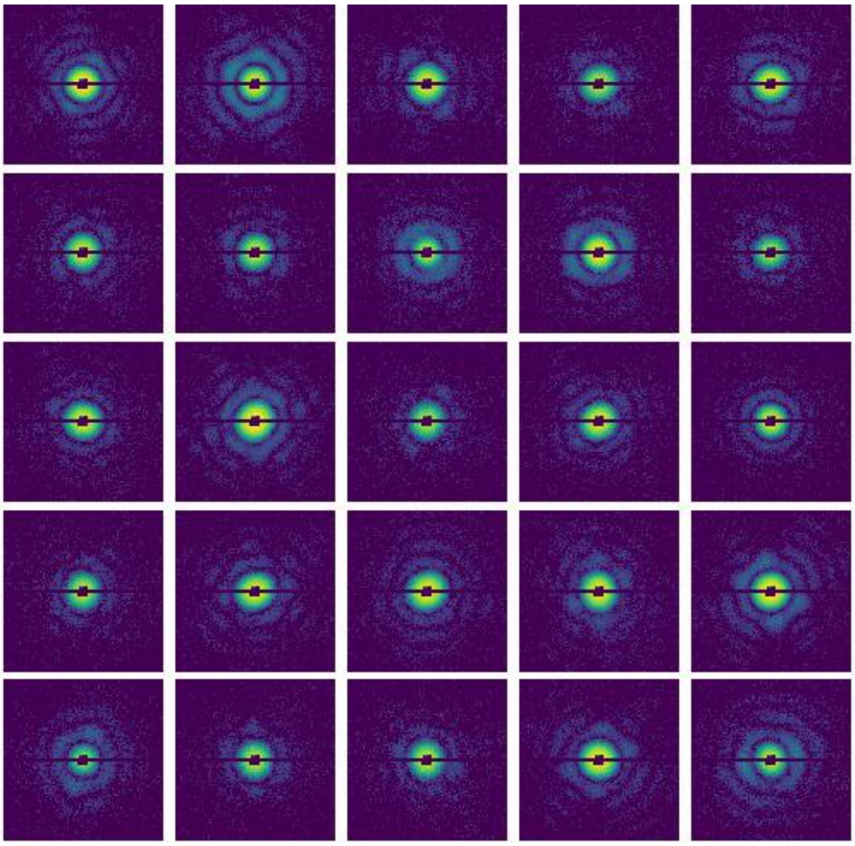

Figure S7 Randomly selected samples of single-particle patterns from classification method (a) CNN, (b) GC, and (c) DM.

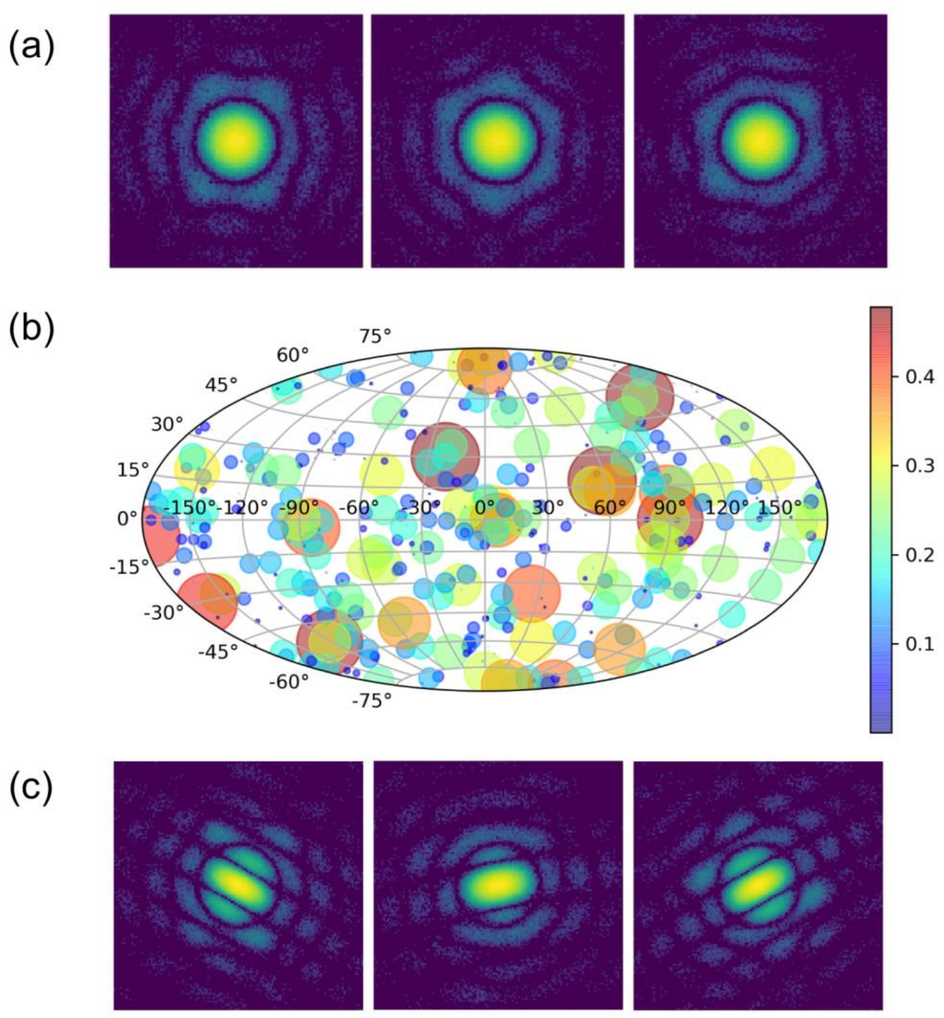

Figure S8 Diffraction simulation of solid icosahedral and orientation recovery. (a) Simulated single-hit patterns from randomly sampled Euler angles; (b) Orientation distributions recovered by EMC algorithm using single-hit patterns; (c) Simulated multiple-hit patterns from randomly sampled Euler angles.

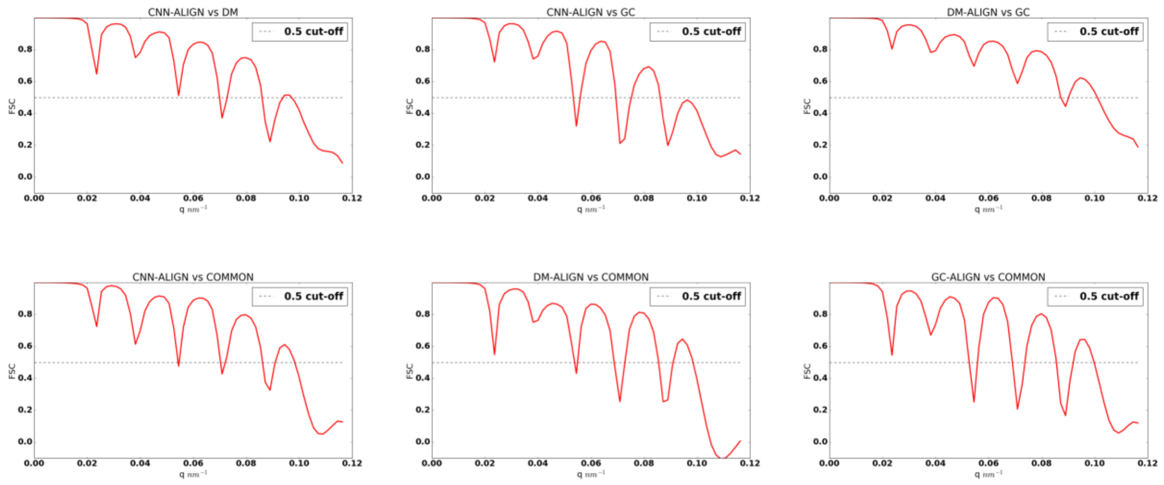

Figure S9 The FSC curves between reconstructed models.
